# Diverse fen plant communities enhance carbon-related multifunctionality, but do not mitigate negative effects of drought

**DOI:** 10.1098/rsos.170449

**Published:** 2017-10-25

**Authors:** Bjorn J. M. Robroek, Vincent E. J. Jassey, Boudewijn Beltman, Mariet M. Hefting

**Affiliations:** 1 Biological Sciences, University of Southampton, Southampton SO17 1BJ, UK; 2 INP, UPS, CNRS, Laboratoire d'Ecologie Fonctionnelle et Environnement (Ecolab), Université de Toulouse, 31062 Toulouse Cedex, France; 3 Ecology and Biodiversity, Utrecht University, Padualaan 8, 3584 CH Utrecht, The Netherlands

**Keywords:** carbon cycling, ecosystem functions, global change, multiple functions, plant functional types, wetlands

## Abstract

Global change, like droughts, can destabilize the carbon sink function of peatlands, either directly or indirectly through changes in plant community composition. While the effects of drought and plant community composition on individual carbon (C) related processes are well understood, their effect on multiple C-related processes simultaneously—multifunctionality—is poorly known. We studied the effect of drought on four C-related processes (net and gross CO_2_ exchange, methane fluxes, and dissolved organic carbon content) in a plant removal experiment. Plant functional type (PFT) removal (graminoids, herbs, *Polytrichum* spp., incl. combinations) negatively affected multifunctionality; most markedly when all PFTs were removed. Our results corroborate a negative drought effect on C-related multifunctionality. Drought reduced multifunctionality, and this reduction was again largest when all PFTs were removed. Our data further indicate that much of these negative drought effects were carried over and maintained from the initial removal treatment. These results suggest that while a high diversity in plant functional types is associated to high C-related multifunctionality, plant community assembly does not drive the ability of peatlands to withstand the negative impacts of drought on multifunctionality. Hence, to safeguard the carbon cycling function in intact peatlands, the effects of climate change on the functional composition of the peatland plant community needs to be minimized.

## 1. Introduction

Global climate change is affecting important ecosystem processes, by altering abiotic and biotic conditions. Climate projections predict, for example, precipitation patterns to become increasingly variable and the frequency of extreme drought to increase for the Northern Hemisphere [1]. Such changes are extremely important for ecosystems that to a certain extent depend on precipitation for their functioning, such as peatlands [2]. As peatlands represent an important sink for atmospheric carbon (C)—they are currently estimated to store about 500 GT of C as peat [3]—the ability of peatlands to act as a carbon sink depends on how the effects of climate change play out on these ecosystems. Much research has been devoted on the role of environmental conditions, including hydrological conditions, on peatland carbon (C) cycling. Overall, consensus exists that the effect of water table drawdown on net peatland C uptake is negative [4,5]. Yet, the responses of individual C-related functions—net ecosystem CO_2_ uptake, ecosystem respiration, the release of methane, or the production and leaching of dissolved organic carbon—to drought can be complex, often with opposite responses for different processes. A drawdown in the water table, for example, generally leads to enhanced vascular plant productivity in peatlands [6,7], although the effect largely depends on the composition of the plant community [8–10]. Clearer is the effect of water table drawdown on carbon uptake by peat mosses; lower water tables impede peat moss productivity [11–13]. The effects of water table drawdown on peatland ecosystem respiration are less straightforward. In some studies ecosystem respiration has been shown to be independent from the water table [14,15], while others report increases in respiration rates with decreasing water tables [4,5,10]. Oppositely, methane fluxes from peat decrease with water table drawdown [16]—despite a potential peak at early drought [4]—and are probably the result of decreased potential methane production and alteration in the microbial communities [17,18]. Lastly, drought has been linked to increased concentrations in dissolved organic carbon (DOC) leaching, mainly through increased decomposition of organic matter [19,20]. In short, while our understanding on the effects of drought on individual C-related processes in peatlands is strong, the effects of drought on multiple, simultaneously occurring processes are poorly understood.

Climate change affects ecosystem processes directly, but also indirectly through alterations in the plant community composition [21], which then affect ecosystem processes. These indirect effects are rather understudied, yet important as changes in the plant community can modulate the negative impact of climate change [22]. In peatland ecology the latter may be because peatland species composition has long been perceived as remarkably stable [23,24]. Historical records, however, link drought to changes in plant community composition [25]. This has led to increased recognition that projected climate change alters species interactions, especially in the temperate climate zone [26]. Further, alterations in the climate or environmental conditions may reduce biological diversity [27,28], the cornerstone for sustaining a variety of ecosystem processes [29,30]. As species typically differ in their functional traits—a morphological, physiological, or chemical characteristic that strongly influences organismal performance—changes in plant species composition or diversity may alter functional trait composition of the community [31]. Depending on the nature of changes in the plant community, climate change may thus lead to an array of feedbacks on peatland processes, including its carbon sink function. In peatlands, the importance of plant community composition for individual ecosystem processes has recently been put forward [12,21,32–35]. Yet, the role of peatland plant community assembly on the ability of peatlands to maintain multiple carbon-related functions—hereafter referred to a C-related multifunctionality—remains unresolved. Moreover, the role of plant functional types on the robustness of C-related multifunctionality to environmental stress, such as drought, is unknown. We aim to bridge this knowledge-gap, and disentangle the effect of vascular plant community composition (using a plant removal approach) and drought C-related multifunctionality in peatlands. We hypothesize (i) that removing plant functional types (graminoids, herbs, non-*Sphagnum* mosses) erodes multifunctionality of carbon-related peatland processes, and that this effect is larger with increasing number of removed plant functional types. Additionally, we hypothesize that (ii) the loss of plant functional types reduces the ability of poor fen communities to withstand the negative effects of drought on multifunctionality.

## 2. Material and methods

### 2.1. Field sampling

The Molenpolder is situated in the Vechtplassen area, a region rich in plant diversity due to the presence of all successional terrestrialization stages from open peat ponds to ombrotrophic peatlands. A total of 24 samples (diameter 22 cm, depth 26 cm) were collected from a poor fen in the Molenpolder, The Netherlands (52°9′7.07′′ N; 5°5′83.18′′ E), in March 2010. The fen is characteristic for the area and of special interest for conservation. It is characterized by a peat soil (1.5–2 m deep) overlain by homogeneous *Sphagnum* spp. (mainly *S. palustre* L., *S. squarossum* Crome) and *Polytrichum commune* Hedw. co-dominated bryophyte layer. The vascular plant community is dominated by the graminoids *Carex echinata* Lam., *Juncus effuses* L., *Juncus acutiflorus* Benth., *Hierochloë odorata* (L.) Britton, Sterns & Poggenb., *Carex hirta* L., *Carex canescens* L. and *Alopecurus geniculatus* Lindh. Ex Scheele, and the herbs *Peucedanum palustre* (L.) Moench, *Lysimachia thyrsiflora* L., *Drosera rotundifolia* L., *Potentilla palustris* (L.) Scop. and *Hydrocotyle vulgaris* L. We sampled in a 10 × 10 m area of a 30 × 75 m mesotrophic fen. This approach was assumed to reduce variance in peat structure, and ensure homogeneity in the vegetation. Extracted mesocosms were, however, never extracted closer than 1 m from each other, while taking care that each mesocosm would contain a selection of species from all plant functional types (*Sphagnum* mosses, *Polytrichum* spp., graminoids, and herbs). Samples, consisting of fen peat and living vegetation, were placed in PVC containers, and are hereafter referred to as mesocosms.

### 2.2. Experimental set-up

All mesocosms were subjected to a four-week acclimatization period in a phytotron (20°C/18°C and 12/12 h day/night, 70% relative humidity (RH), 400 ppm CO_2_, 200 µmol PAR m^−2^ s^−1^ light intensity), where we maintained water tables at field condition (1–2 cm below the bryophyte surface), using an artificial rainwater solution [36]. After this acclimatization period, plant communities were experimentally manipulated. All mesocosms were randomly allocated to one of the following treatments (*n* = 4): controls (C), graminoid removal (–Gram), herb removal (–Herb), *Polytrichum* removal (–Poly), graminoid + herb removal (–Gram & Herb), and graminoids + herb + *Polytrichum* removal (–Gram & Herb & Poly). Due to experimental limitations we did not remove *Polytrichum* mosses in combination with graminoid or herb removal. Further, *Sphagnum* mosses were never removed as they are considered key to the functioning of the ecosystem. For the vascular plants, removal was realized by clipping the aboveground biomass flush to the moss layer. *Polytrichum* was removed by pulling all individuals from the mesocosms. This was done cautiously to minimize perturbation in the peat soil. The control mesocosms were left unchanged in species composition, yet to control for potential effects of the treatment we selectively removed 5–10% of the vascular plant and *Polytrichum* cover (balanced over the present plant functional types (PFTs) and based on visual comparison of the effect of the clipping on the plant cover in removal mesocosms). As a result of these treatments, we removed 0.77 ± 0.1 g in the control mesocosms, 4.55 ± 1.4 g in the graminoids removal mesocosms, 0.24 ± 0.07 g in the herb removal mesocosms, 13.5 ± 4.9 g in the *Polytrichum* removal mesocosms, 3.53 ± 0.4 g in the graminoids & herb removal mesocosms, and 9.76 ± 2.6 g in the graminoid & herb & *Polytrichum* removal mesocosms. Throughout the experiment, treatments were reinforced by regular removal of regrowth.

After plant removal, mesocosms were left to acclimatize over a four-week period (*post-clipping acclimatization*). Previous research with ombrotrophic bog mesocosms has shown this time to be sufficient to minimize the effect of decaying roots on ecosystem respiration [12]. During the *post-clipping acclimatization* period, mesocosms were watered two times per week to maintain field conditions. Following the *post-clipping acclimatization*, a 25 day *drought* period was initiated. This period commenced by draining all water from the mesocosms, and a full stop of the watering regime.

### 2.3. Ecosystem function measurements

Closed transparent flux chambers (diameter 20 cm, height 29 cm, fitted with a circulating fan) were placed over the mesocosms to measure CO_2_ and CH_4_ fluxes, using a photoacoustic multi-gas analyser (Innova Bruel and Kjær BK 1302) connected to a multipoint sampler (CBISS MK2, 4-channel, CBISS Ltd., England). Chamber measurements comprised five succeeding sampling points with an 8 min interval. Net ecosystem exchange (NEE) was measured weekly during the *acclimatization* and the *post-acclimatization* period, to enable calculating reliable initial values (see below). To assess the effect of drought, NEE was measured at the first and last day of the *drought* period. Ecosystem CO_2_ respiration (R_ECO_) was measured in parallel, using a darkened chamber. Further, pore-water samples were extracted from all mesocosms at the time of gas measurements using Rhizon soil moisture samplers (type MOM, pore size 0.1 µm, Eijkelkamp, Giesbeek, NL). Pore-water samples were stored in glass vials in the dark at 4°C and were analysed for dissolved organic carbon (DOC) within two weeks after collection, using a Skalar SANPLUS segmented flow analyser (Skalar analytical b.v. Breda, NL). As DOC is highly mobile in the peat, we assume this part of the peat carbon pool to represent the labile carbon pool, vulnerable to leaching and able to enhance decomposition of organic matter by stimulating the microbial community.

NEE was calculated from the change in concentration in the chamber headspace with time, using an exponential nonlinear function [37]. CH_4_ fluxes and R_ECO_ were calculated using linear regression of gas concentrations in the chamber headspace over time. Gross ecosystem production (GEP) was calculated as the sum of NEE and R_ECO_. The ecological sign convention was used for the CO_2_ (NEE and GEP) and CH_4_ data, so that positive values indicate a sink function, while negative values indicate a source function of the ecosystem. High concentrations of DOC, which is the product of decomposition and plant exudation, were seen as a negative function, as these DOCs can be lost from the ecosystem. To maintain directional change comparable to the other functions we calculated inversed DOC concentrations (i.e. low inversed values = high DOC concentrations).

### 2.4. Ecosystem multifunctionality

**Figure 1.**
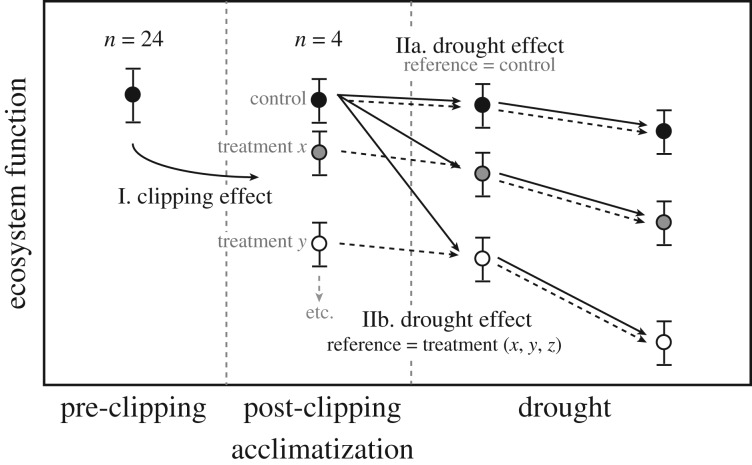
Schematic set-up of the data analyses. The effect of clipping on each ecosystem function (I. clipping effect) was calculated as the difference in ecosystem function before clipping (mean of all values, *n* = 24) and after clipping. *z*-values were calculated after standardization by the pre-clipping mean and standard deviation. The effect of drought was calculated in two different ways. First (IIa), the effect of drought for each plant removal treatment on each ecosystem function was calculated as the difference in the respective function during drought and the post-clipping acclimatization control values. Hence, for each treatment *z*-values were calculated after standardization by the post-clipping acclimatization control. In the second approach (IIb), instead of using the post-clipping acclimatization control as a reference, the post-clipping acclimatization ecosystem function values for each corresponding treatment were used.

The overall effects of plant removal and drought on C-related processes were tested using a multifunctionality approach [38]. Here, multifunctionality is defined as a single metric (*z*-score) describing the overall function of net CO_2_ and CH_4_ ecosystem exchange, gross ecosystem production, and the production and leaching of dissolved organic carbon. We calculated multifunctionality in a multiple-step approach. First, per mesocosms data for each process were averaged for the four time points during the *acclimatization* and *post-clipping acclimatization* period. This step was necessary to reduce the variance in the ecosystem process values, hence provide a robust value for further analyses. Next, to calculate the effect of plant functional type removal on ecosystem processes per treatment, we standardized the process values by the global mean and standard deviation (i.e. the mean and standard deviation of all mesocosms, *n* = 24) of the *pre-clipping* ecosystem process (figure 1). To assess the effect of drought on ecosystem processes we used two approaches (figure 1). In the first approach (figure 1, IIa), we standardized ecosystem process during the drought period for each treatment by the mean and standard deviation of the *post-clipping acclimatization* control values. In the second approach (figure 1, IIb), ecosystem process values during drought for each treatment were standardized by the mean and standard deviation of the *post-clipping acclimatization* values of the corresponding treatment. These approaches differ in that the first approach takes the non-clipped control values as a reference, while the second approach tests for the effect of drought irrespective of apparent effects of clipping on ecosystem processes. Subsequently, the *z*-scores were used as an index of ecosystem multifunctionality [39]. To remain close to natural processes and differences in the individual process rates, and hence be relevant to land managers, we did not scale individual process values. Negative *z*-scores indicate low multifunctionality, while positive *z*-scores indicate high multifunctionality.

### 2.5. Data analyses

The effects of the plant functional type removal treatments on individual ecosystem processes and multifunctionality was tested using generalized linear models (GLMs) assuming Gaussian data distribution. As the amount of biomass removed may explain part of the effects, we initially tested different models: one that excluded, and one that included biomass removed (g) as a covarying factor. Using the selMod function in the *pgirmess* package in R, we selected the model with the smallest corrected Akaike information criterion (AICc). This model was then subjected to analysis of variance, using the ANOVA function and the F statistic in the *stats* package, followed by a Tukey multicomparison test to highlight differences between treatments. The effect of clipping and drought (for both standardization approaches) on multifunctionality was analysed in a similar manner. All analyses were performed with the software R 3.2.3.

## 3. Results

### 3.1. Plant removal effect on individual ecosystems functions

**Figure 2.**
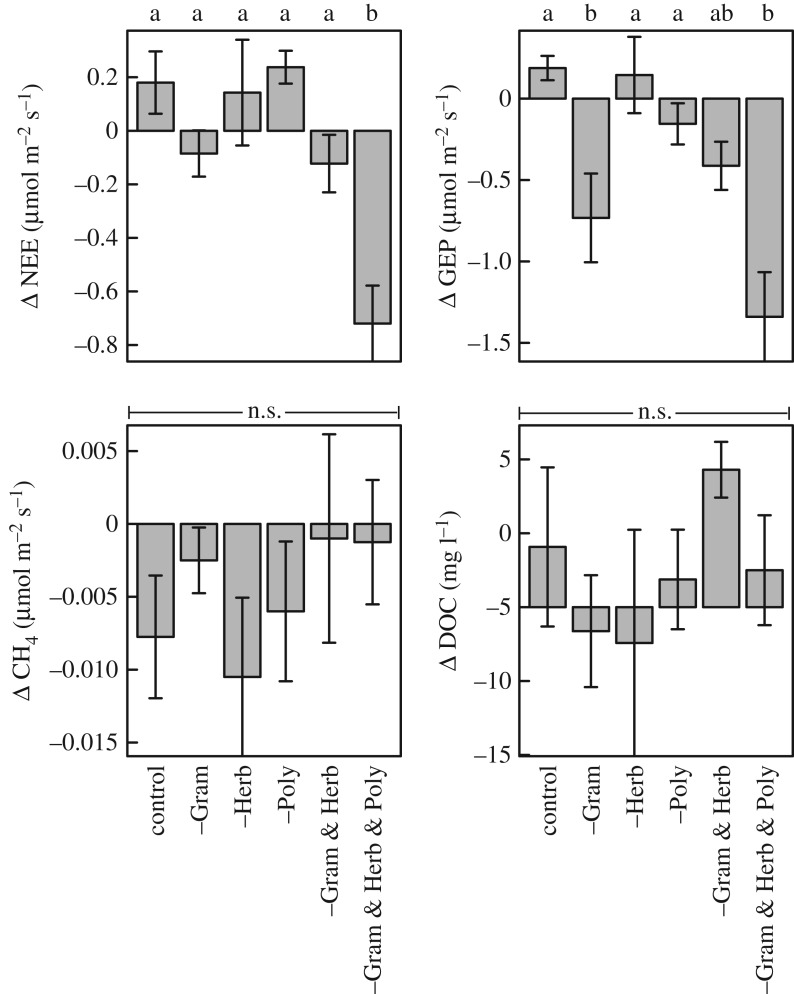
The effect of the removal of plant functional types on net ecosytem CO_2_ exchange, gross ecosystem production, CH_4_ production, and the dissolved organic carbon (DOC) content in the pore water. Bars represent the change in the four carbon-related processes after plant biomass removal. Different letters indicate significant difference between PFT removal treatments (Tukey's multi-comparison test, *p* ≤ 0.05). We tested the effect of biomass removal on our model outcomes, see table 1.

Plant functional type removal affected net ecosystem carbon exchange (NEE) and gross ecosystem production (GEP), but not methane (CH_4_) fluxes and pore water dissolved organic carbon (DOC) concentrations (table 1 and figure 2). Notably, removing all vascular plant functional types from the mesocosms caused NEE to decrease dramatically. GEP decreased upon plant removal in those treatments that included the removal of graminoids (i.e. –Gram, –Gram & Herb, –Gram & Herb & Poly), and could be partly explained by the loss of biomass (table 1 and figure 2). While not significant (table 1), the removal of more than one plant functional type seemed to lower methane production by the peat (figure 2).

**Table 1.** (*a*) Results of the model testing, comparing the power of two models—one with only the plant removal treatments, one with both the removal treatment and the amount of biomass removed as factors—in explaining the change in ecosystem CO_2_ net exchange (NEE), gross ecosystem production (GEP), net methane (CH_4_) flux, and the content of dissolved organic carbon, before and after plant removal. LL_max_ = maximized log-likelihood of the model, AIC = Akaike Information criterion, AIC_c_ = corrected AIC. (*b*) Results of analysis on variance (ANOVA) on the most significant model, i.e. the model with the lowest AIC_c_.

(*a*)				
ecosystem process	model	LL_max_	AIC	AIC_c_
NEE	∼treatment	2.5	9.1	16.06
	∼treatment + biomass removed	3.3	9.4	18.95
GEP	∼treatment + biomass removed	–5.3	26.6	36.21
	∼treatment	–9.0	32.0	39.00
CH_4_-flux	∼treatment	80.3	–146.7	–139.69
	∼treatment + biomass removed	80.3	–144.7	–135.10
DOC	∼treatment	–84.2	182.4	189.44
	∼treatment + biomass removed	–83.9	183.8	193.36
(*b*)				
	factor	*F*-value	*p*-value	
NEE	treatment	7.9	≤0.001	
GEP	treatment	10.5	≤0.001	
	biomass removed	6.1	0.024	
CH_4_-flux	treatment	0.6	0.686	
DOC	treatment	0.8	0.547	

### 3.2. Plant removal effect on carbon-related multifunctionality

**Figure 3.**
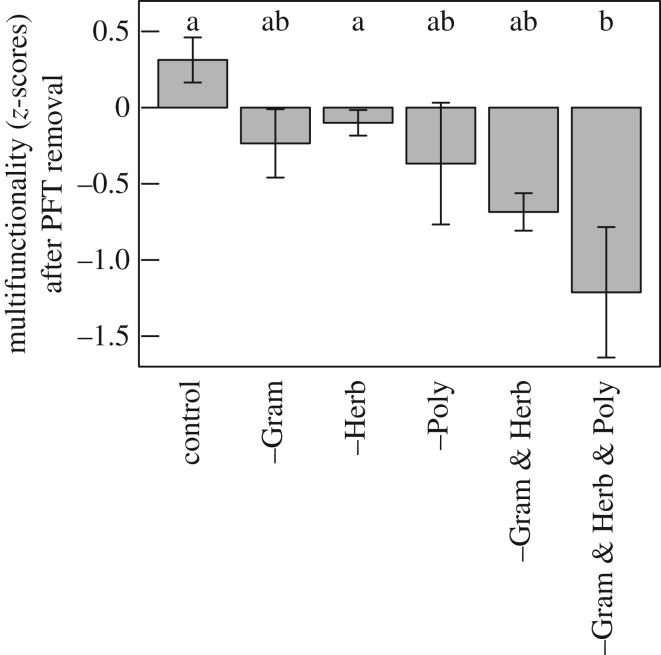
Ecosystem multifunctionality after biomass removal in relation to plant functional type (PFT) removal treatment. Multifunctionality was calculated as the mean *z*-value calculated from the standardized (overall pre-clipping mean and standard deviation, figure 1) ecosystem values. Different letters indicate significant difference between PFT removal treatments (Tukey's multi-comparison test, *p* ≤ 0.05).

Multifunctionality in carbon-related ecosystem processes was in general eroded when plant functional types were removed (figure 3). The removal of graminoids, herbs, *Polytrichum*, and graminoids & herbs resulted in a non-significant decrease (175, 132, 217, and 319%, respectively) in multifunctionality. The removal of all vascular plant functional types enhanced this trend (figure 3, 588%), and in line with the aforementioned was mainly due to the decrease (*p* ≤ 0.05) in NEE and GEP (figure 2). The removal of 5–10% of the plant cover without changing the community composition, i.e. the control plots, showed an increase in multifunctionality, underpinning the negative effect of the loss of PFTs on C-related multifunctionality.

### 3.3. The effect of drought on ecosystem multifunctionality

**Figure 4.**
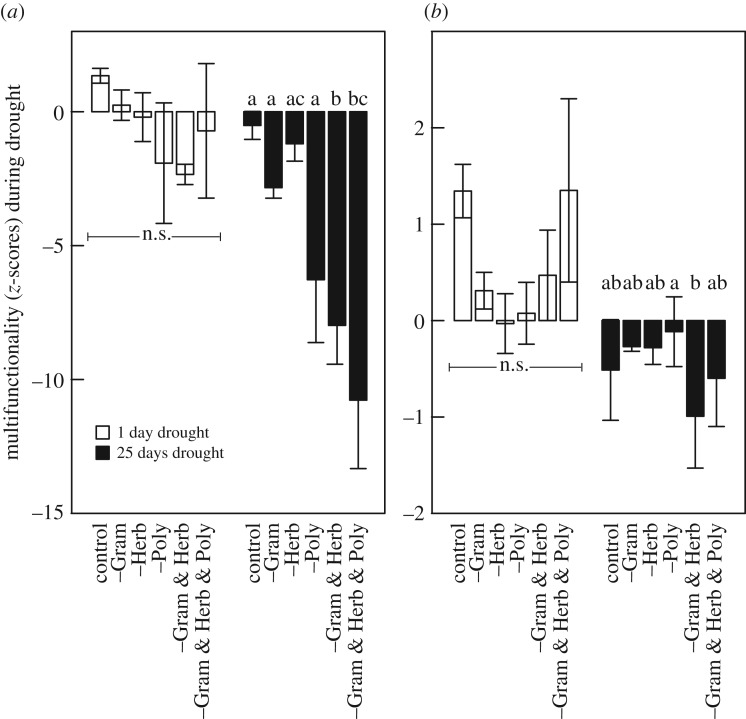
Ecosystem multifunctionality after the initiation of an experimental drought in relation to plant functional type (PFT) removal treatment. C-related multifunctionality was calculated in two ways: First, (*a*) as the mean *z*-value calculated from individual ecosystem values standardized by mean and standard deviation values of the post-clipping acclimatization control treatments (figure 1, IIa drought effect); second, (*b*) as the mean *z*-value calculated from individual ecosystem values standardized by mean and standard deviation values of the corresponding post-clipping acclimatization PFT treatments (figure 1, IIb drought effect). The relationships between PFT removal and each individual ecosystem function over the experimental period are shown in electronic supplementary material, figure S1. Different letters indicated significant difference between PFT removal treatments (Tukey's multi-comparison test, *p* ≤ 0.05; n.s., not significant), analysed separately for the two times after initiation of drought.

When compared to the *post-clipping acclimatization* values of the control, i.e. the unchanged communities, C-related multifunctionality was unaffected directly after the initiation of drought, but eroded after the 25 day drought period (figure 4*a* and table 2). Within-time analysis (*t* = 1, *t* = 25) indicates no plant removal effect on multifunctionality directly after the initiation of drought owing to, in part, the loss of biomass (table 2). After the 25 day drought period, multifunctionality was eroded (*p* ≤ 0.05) when more than one plant functional types were removed (figure 4*a* and table 2). The negative effects of PFT removal in C-related multifunctionality are likely ‘carried over’ from decreased *z*-values prior to the initiation of drought (figure 4*a*; electronic supplementary material, figure S1). To elucidate this, we performed a similar test but now compared multifunctionality values during drought to the *post-clipping acclimatization* values of the corresponding treatment (intra-treatment comparison, figure 1). Multifunctionality one day after the initiation of drought increased (non-significant) irrespective of plant functional type removal, but decreased after 25 days of drought (figure 4*b*). These results underpin a strong eroding effect of drought on C-related multifunctionality, but also attest for an absence of a role of plant community assembly thereon (figure 4*b* and table 2).

**Table 2.** Results of repeated measures analysis of variance (RM–ANOVA), testing the effect of plant functional type removal, incl. biomass removed (co-variable) on the *z*-values during drought. The two different ways of testing refer to the method of calculating multifunctionality (see text in Material and methods, figure 1). n.s., non-significant.

	df_num_,df_den_	*F*-value	*p*-value
*drought effect IIa on multifunctionality*			
overall			
time	1,46	29.7	≤0.001
treatment	1,41	7.0	≤0.001
time × treatment	1,35	2.8	≤0.05
biomass removed	1,40	9.0	≤0.01
*t* = 1			
treatment	1,18	0.9	0.530
biomass removed	1,17	0.3	0.569
*t* = 2			
treatment	1,18	13.9	≤0.001
biomass removed	1,17	20.8	≤0.001
*drought effect IIb on multifunctionality*			
overall			
time	1,46	16.4	≤0.001
treatment	1,41	0.8	0.582
time × treatment	1,36	1.6	0.184
biomass removed			n.s.

## 4. Discussion

Fens have been intensively studied in the context of nitrogen and phosphorus pollution [40–42] and vegetation succession and distribution [43,44]. Additionally, the effects of climate change driven alterations in plant community assembly on poor fen ecosystem processes are well understood [21]. The combined effects of plant functional types on the overall performance of these ecosystems, and on the ecosystems' ability to sustain these functions, to our best of knowledge, however, remain elusive. In grassland ecosystems, functional identity and diversity of the plant community are important drivers for ecosystem multifunctionality [45–47]. Our study does not necessarily reconcile these findings. We suggest that the less pronounced effects of plant functional type (PFT) removal on C-related multifunctionality in our study are explained by opposing responses in individual processes that balance the mean performance over the four carbon-related processes [48]. To illustrate, a decrease in NEE upon PFT removal seems to be counteracted by a reduction in methane fluxes; the latter most likely caused by reduced methanogenic activity through decreased input of labile carbon [18,34,49]. While PFT removal decreases gross C uptake (GEP), depending on the nature of the PFT, the related decrease in biomass results in lower maintenance respiration. This explains, in part, the close resemblance in patterns of NEE and GEP. Nevertheless, and despite the absence of pronounced statistical differences in multifunctionality after PFT removal, the removal of PFTs from the communities always caused multifunctionality to shift from positive to negative. Moreover, the removal of single PFT resulted in a decrease in the simultaneous performance of the four C-related processes. These results reflect general understanding that a high level of diversity is needed to sustain multifunctionality [50–52]. Most likely, plant–microbe interactions play an important role in explaining our results, as changes in the peatland plant community are repeatedly reported to be reflected in the microbial community [34,53,54]. Decreased biodiversity, and in our case the loss of plant functional types, may even reduce the functional diversity of the microbial community [55]. Such reduction of microbial functional diversity may in turn further erode ecosystem multifunctionality [46].

Multifunctionality is an average measure that considers a set of ecosystem processes simultaneously, and should be interpreted with care. If, for example, different functions (i.e. carbon, nitrogen and phosphorus cycling, biodiversity provisioning, etc.) are considered, opposite function may moderate the value of multifunctionality, making ecological interpretation difficult without assessing individual functions. We argue, however, that describing multifunctionality from simultaneous processes that contribute to a single function—C-related multifunctionality—is very powerful as it allows a holistic assessment of the ecosystem function. In other words, while the individual processes that underlie an ecosystem function are important, how these individual processes play out simultaneously—multifunctionality—provides more understanding on the overall status of the particular ecosystem function. Our results highlight that although the influence of plant community assembly on C-related multifunctionality seems to level out due to contrasting effects on individual processes, the loss of individual plant functional types had an overall negative effect on these processes. This then resulted in a slight, though non-significant, decrease in the overall carbon cycling function of the poor fen system.

We calculated the effect of drought on C-related multifunctionality in two ways, one where *pre-drought* ecosystem function values of the undisturbed control mesocosms served as a reference, and one where the values during drought were compared to values *pre-drought* from the same treatment. Both results show that drought exacerbates the erosion in poor fen C-related multifunctionality. The first approach shows that, with almost one month of drought, the removal of more than one plant functional types strongly reduces ecosystem multifunctionality. The second approach, where within-treatment effect of drought on multifunctionality was assessed, does not show such effect. This would mean that *pre-drought* differences in multifunctionality, caused by PFT removal, while exacerbated during drought, persist during drought. In a previous study on mesocosms from a *Sphagnum*-dominated bog, it has been shown that removing vascular plant functional types decreased net carbon uptake but not the robustness of the ecosystem as a carbon sink to withstand drought [12]. Our results corroborate these findings, and underpin earlier findings on the importance of the peat moss community and the peat matrix for sustaining the functioning of the ecosystem. In other words, non-*Sphagnum* plant functional types are largely responsible for the magnitude of the peatland carbon sink function, but play less a role in sustaining that function during environmental perturbation like drought.

The results from our study are important, as climate change is known to alter the composition of peatlands, with pronounced shifts between vascular plants and peat mosses, as well as between plant functional types within these larger groups [7,56]. Increased temperature and drought occurrences can increase vascular plant growth leading to a decrease in peat moss growth [21]. Subsequently, shifts in the competitive balance between plant functional types may result in the loss of key plant functional types, weakening the carbon sink function of peatlands [21,57]. In this study, *Sphagnum* mosses were never removed as they are a crucial part of the ecosystem in *Sphagnum*-dominated peatland [58], and removing them from the system would result in a non-viable ecosystem [32]. In the light of our results, the loss of single vascular plant functional types from fen ecosystems only marginally affects the ability of these ecosystems to sustain multiple carbon-related functions. Oppositely, our results indicate that a diverse plant functional type composition is most effective in sustaining C-related multifunctionality. Results from a recent study show that the protection of current carbon stocks is important in order to slow down the rate of increases in atmospheric CO_2_ [59]. Our study reconciles with such statement; protecting the diversity in plant communities in northern peatlands, while not increasing the robustness of these systems to projected drought, increases the overall C-sink function of these systems.

## Supplementary Material

Drought effect on carbon related processes

## Data accessibility

Datasets are deposited at the Dryad Digital Repository (http://dx.doi.org/10.5061/dryad.g1pk3) [60].
