# Supplementary material for: Diverse fen plant communities enhance carbon-related multifunctionality, but do not mitigate negative effects of drought
Source: R Soc Open Sci. 2017 Oct 25;4(10):170449. doi: 10.1098/rsos.170449 (PMC5666246; doi:10.1098/rsos.170449)
Supplement: Drought effect on carbon related processes [file rsos170449supp1.docx]

Supplementary information to:

Robroek, Jassey, Beltman & Hefting. Diverse fen plant communities enhance carbon-related multifunctionality, but do not mitigate negative effects of drought. Royal Society Open Science

**supplementary figure 1**. The effects of plant functional type removal on net ecosystem CO2 exchange, Gross ecosystem production, CH4 production, and the Dissolved Organic Carbon (DOC) content in the pore water, just before (-1), just after (1) and 25 days after the initiation of an experimental drought. C = control treatment, G = graminoids removed, H = herbs removed, P = *Polytrichum spp.* removed, GH = graminoids & herbs removed, GHP = graminoids, herbs & *Polytrichum spp.* removal.

**Data accessibility.** Datasets are deposited at the Dryad digital repository: [http://dx.doi.org/10.5061/dryad.5b352](http://dx.doi.org/10.5061/dryad.5b352).
